# The evidence informing the surgeon's selection of intraocular lens on the basis of light transmittance properties

**DOI:** 10.1038/eye.2016.266

**Published:** 2016-12-09

**Authors:** X Li, D Kelly, J M Nolan, J L Dennison, S Beatty

**Affiliations:** 1 Pharmaceutical & Molecular Biotechnology Research Centre, Department of Chemical & Life Sciences, Waterford Institute of Technology, Waterford, Ireland; 2 Nutrition Research Centre Ireland, Macular Pigment Research Group, School of Health Science, Waterford Institute of Technology, Waterford, Ireland; 3 Institute of Vision Research, Whitfield Clinic, Waterford, Ireland

## Abstract

In recent years, manufacturers and distributors have promoted commercially available intraocular lenses (IOLs) with transmittance properties that filter visible short-wavelength (blue) light on the basis of a putative photoprotective effect. Systematic literature review. Out of 21 studies reporting on outcomes following implantation of blue-light-filtering IOLs (involving 8914 patients and 12 919 study eyes undergoing cataract surgery), the primary outcome was vision, sleep pattern, and photoprotection in 9 (42.9%), 9 (42.9%), and 3 (14.2%) respectively, and, of these, only 7 (33.3%) can be classed as high as level 2b (individual cohort study/low-quality randomized controlled trials), all other studies being classed as level 3b or lower. Of the level 2b studies, only one (14.3%) found in favor of blue-light-filtering IOLs *vs* ultraviolet (UV)-only filtering IOLs on the basis of an association between better post-operative contrast sensitivity (CS) at select frequencies with the former; however, that study did not measure or report CS preoperatively in either group, and the finding may simply reflect better preoperative CS in the eyes scheduled to be implanted with the blue-light-filtering IOL; moreover, that study failed to measure macular pigment, a natural preceptoral filter of blue-light, augmentation of which is now known to improve CS. In terms of photoprotection, there is no level 2b (or higher) evidence in support of blue filtering IOLs *vs* UV-only filtering IOLs. On the basis of currently available evidence, one cannot advocate for the use of blue-light-filtering IOLs over UV-only filtering IOLs.

## Introduction

Cataract surgery and intraocular lens (IOL) implantation is the most commonly performed surgical procedure worldwide,^1^ and the need for such surgery is likely to rise because of increasing longevity.^2^ Commercially available IOLs can vary in terms of material (polymethylmethacrylate, polymers, silicone or acrylic),^3, 4^ hydrophobicity (hydrophobic *vs* hydrophilic),^5^ sphericity (aspheric *vs* spheric)^6^ and light-filtering properties. In this paper, we review the evidence germane to the putative and relative risks and/or benefits of implanting IOLs that filter visible short-wavelength light (ie, <500 nm), referred to as blue-light-filtering IOLs for the purpose of this review.

In this article, we grade each study according to the quality of evidence it represents, on the basis of the Oxford (UK) Centre for Evidence-Based Medicine (CEBM) criteria, described below:

1a. Systematic review (homogeneous) of randomized controlled trials (RCT) with narrow confidence intervals: RCTs are studies where the participants are randomly allocated to one or other of the different interventions under investigations, and the greater the sample size, the reduced likelihood of bias;

1b. Individual RCT with narrow confidence interval.

2a. Systematic review of (homogeneous) cohort studies.

2b. Individual cohort study/low-quality RCT.

3a. Systematic review of (homogeneous) case-control studies.

3b. Individual case-control studies.

4. Case series, low-quality cohort, or case-control studies.

5. Expert opinions without explicit critical appraisal, or based on physiology, bench research or first principles.

## **The visible and near-visible spectrum**

The visible and near-visible spectrum represents a small proportion of the total electromagnetic spectrum, and comprises wavelengths (*λ*) ranging from 200 to 780 nm. This includes ultraviolet (UV) radiation, visible light (400–780 nm), and some short-wavelength infrared radiation.^7, 8^ The cornea, vitreous, and aqueous transmit wavelengths >300 nm, and it rests on the crystalline lens to absorb UV radiation, which it does in an age-dependent manner (ie, optical lens absorption increases with age).^9, 10, 11^

### Vision and the visible spectrum

Vision depends on photoreceptive cells adapted to the lighting conditions, and may be classed as photopic (vision under well-lit conditions; mediated by short-wavelength [S], middle-wavelength [M], and long-wavelength [L] cones; maximum efficacy at 683 lm/W at 555 nm; luminance levels of 10 to 10^6^ cd/m^2^), scotopic (vision under very poorly-lit conditions; mediated exclusively by spectrally insensitive rods; luminance levels of 10^−2^–10^−6^ cd/m^2^) and mesopic (under low, but not quite dark, lighting conditions; mediated by rods and cones; luminance levels of 10^−2^–10 cd/m^2^).^12^ Of note, the terms ‘scotopic' and ‘mesopic' are not interchangeable, as rods contribute solely to scotopic vision whereas mesopic vision is subserved by rods and cones.

The impact of dark adaptation, that is, switching from cones to rods to process light, is known as the Purkinje shift, and results in a shift of the peak luminance sensitivity of the human eye from the red end toward the blue end of the spectrum at low illumination levels,^12^ such that blue light is responsible for 35% of aphakic scotopic vision compared with only 7% of photopic vision.^13, 14^

### Central vision

Central vision is mediated by the central macula, a region of the retina ~3 mm in diameter centered at the *fovea centralis* (positioned at 0° eccentricity) and slightly larger than the region that contains the macular carotenoids (4–6° in diameter).^15^ To subserve its function in terms of fine detail vision, the macula is dominated by cones, which are stimulated by high-intensity light. The three types of cones (S-, M-, and L-) each contain different opsins that alter the spectral absorption properties of the photopigments, each therefore preferentially responding to short-, middle-, and long-wavelength visible light, respectively.^16^ The red/green (R/G) color system is served by the L- and M-cones, whereas the blue/yellow (B/Y) color system is served by the S-cones and a combination of the L- and M-cones.^17, 18^ Studies have shown good evidence that the B/Y channel has adaptation mechanisms to compensate for short-wavelength light loss (due to macular pigment (MP) and the crystalline lens), whereas this is not the case with the R/G channel and has not been shown for rods.^19, 20^

### Visible short-wavelength light

For the purpose of this review, visible short-wavelength light refers to wavelengths between 400 and 500 nm, and includes violet (400–440 nm) and blue (440–500 nm).^14^

### Visible short-wavelength light and central vision

#### Blue light and chromatic aberration

Short-wavelength light is deleterious to image formation at the central fovea because of the consequences of chromatic aberration (CA) and light scatter. CA (specifically longitudinal CA) refers to the wavelength-dependence of the refractive power of the eye, whereby (in an emmetropic state) green wavelengths (520–570 nm) are focused at the foveal plane, but blue light is myopically defocused by ~1.2 diopters, resulting in a blurred image.^21^ Transverse CA results from oblique incident light waves being focused in the same focal plane, but not along the optical axis, thus resulting in a blue haze surrounding the object being viewed.^22, 23^ CA adversely affects the ability to discern the foreground from the background within the field of view (known as contrast sensitivity, or CS),^24, 25, 26^ although there is some evidence to suggest that the eye's optical imperfections go some way to attenuating the effects of longitudinal CA.^27^

#### Blue light and light scatter

Light scatter, which occurs both internal and external to the eye, is responsible for veiling luminance and glare disability.

Straylight is the consequence of intraocular light scatter, and is classed as forward scatter (by the cornea and lens, each of these structures contributing to ~30% of intraocular light scatter within the young, healthy, Caucasian eye) and sideward scatter (by the fundus, accounting for ~40% of intraocular light scatter, and which decreases with increasing eccentricity, such that such sideward scatter in the peripheral retina is only half that occurring at the fovea).^28, 29^ The wavelength-dependence of intraocular light scatter remains a matter of debate, with some studies suggesting that visible short-wavelength (blue) light is subject to a greater degree of intraocular light scatter than are longer visible wavelengths.^9, 30, 31^

External to the eye, light waves are reflected and diffracted by a variety of particles suspended in the atmosphere, and such particles include oxygen and nitrogen, haze aerosols, fog, mist, clouds, amongst others. When light is incident on particles smaller than the wavelength of light, Rayleigh (ie, non-directional) scattering occurs, where the degree of scattering is proportionate to the inverse fourth power of the wavelength. Therefore, short-wavelength blue light is scattered much more than light of longer wavelengths.^32^ When particle size is greater than ~0.1*λ*, Mie scattering occurs.^33^ In Mie scattering, particles randomly reflect the waves of light. Both Rayleigh and Mie scattering contribute to veiling luminance, the former being more important and wavelength-dependent, rendering the scatter of blue visible light the predominant cause of veiling luminance and glare disability.^32, 34^

The consequences of veiling luminance are best illustrated by the experience of someone attending a barbecue on a sunny, summer afternoon, when the embers of the coal appear gray, and cannot be discerned one from the other. Several hours later, at twilight, and when there is little visible light (and therefore inconsequential light scatter), the embers are clearly discernible in spite of unchanged luminances of the embers. This occurs because the just-noticeable-differences for discriminability of objects of differing luminance are increased in the presence of visible light scattered across the retina (veiling luminance).^32, 35^ Of note, the adverse impact of veiling luminance is compounded in the presence of environmental light that is too intense or variable across the visual field (a phenomenon known as glare disability), reflected in the visual experience of someone on a sailing boat on a bright summer's day.^36, 37^ Importantly, under such conditions, loss of CS is greater in dim than in bright light environments (eg, strong oncoming headlights whilst driving at night) because rods need greater contrast differences for target detection than do cones (~20 *vs* 1%, respectively).^36^

Finally, it should be borne in mind that the central fovea does not contain S-cones, and therefore blue visible light cannot contribute to high-frequency spatial vision.^38, 39^

### Light-filtering properties and visual consequences of macular pigment

**Figure 1:**
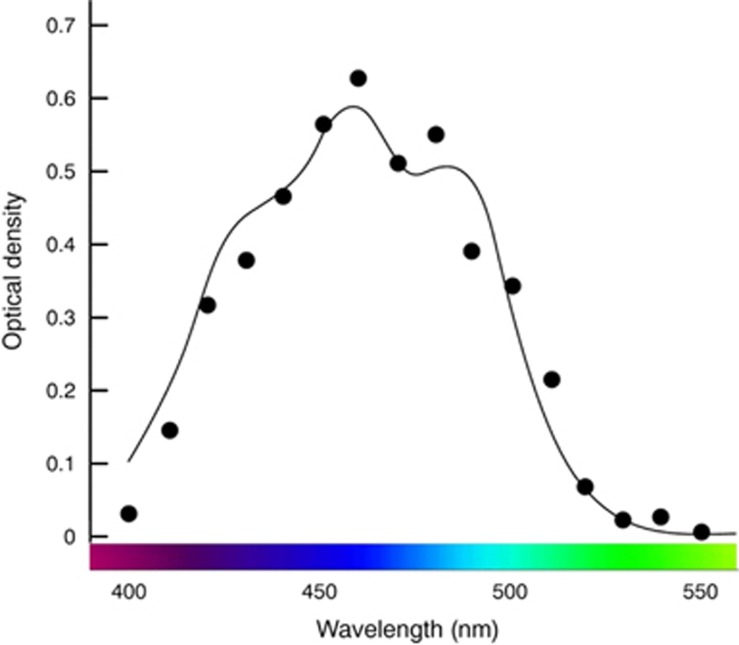
The absorbance spectrum of MP peaks at 460 nm. An average amount of MP (0.40 optical density units) filters out ~40% of blue light incident on the macula.^40, 41^

**Figure 2:**
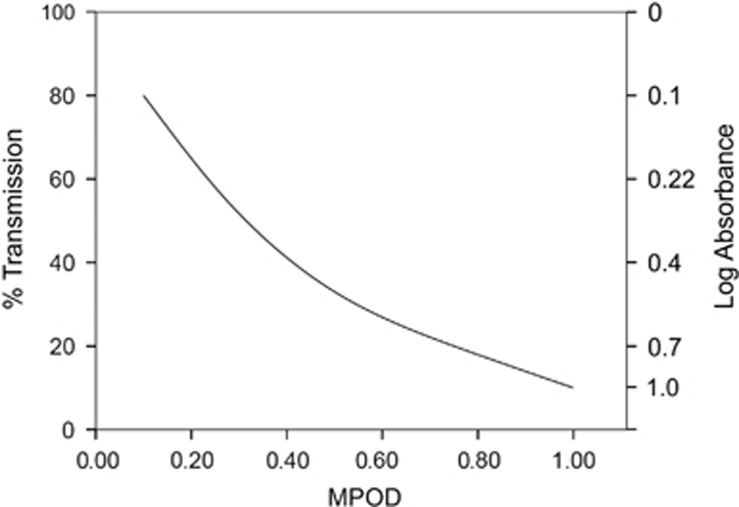
The percent transmission and log absorbance of 460 nm light by MP, with respect to MPOD. Transmission of visible short-wavelength (blue) light decreases substantially with increasing MPOD.^32^

Preceptoral filtration of short-wavelength visible light at the fovea is important for optimal central vision, and this is achieved naturally by the selective accumulation of three carotenoids (lutein [L], zeaxanthin [Z], and *meso*-zeaxanthin [MZ]) at this tissue, collectively referred to as MP.^34^ The absorbance spectrum of MP peaks at 460 nm (blue visible light), and an average amount of MP (0.40 optical density units) filters out ~40% of blue light incident on the macula (Figure 1).^40, 41^ Wooten and Hammond reported that transmission of visible short-wavelength (blue) light decreases substantially with increasing MP optical density (MPOD), so that 0.10 MPOD units would transmit ~80% of light at 460 nm, 0.50 MPOD units would transmit ~33% of light at 460 nm, and 1.00 MPOD units would transmit ~10% of light at 460 nm (Figure 2).^32^

Accordingly, the investigators hypothesized that the resulting decrease in luminance of this short-wavelength light due to MP absorbance would improve contrast between a background (consisting of blue haze) and a target, thereby increasing visual range and improving discernibility of a target's low-contrast internal details in a way that is proportionate to MPOD. In practical terms, therefore, an average amount of MP (0.5 OD) would increase visual range by 18.6%, whereas a high level of MP (1.0 OD) would increase visual range by 30%. Equally, and in terms of CS, a target that is discriminated 50% of the time in the absence of MP (0.0 OD) is discerned 88% of the time in the presence of an average amount of MP (0.5 OD) and almost 100% of the time for a high amount of MP (1.0 OD).^32^ Indeed, the visual benefits of MP are consistent with the results of clinical trials in diseased^42, 43, 44, 45^ and non-diseased eyes.^34, 46, 47, 48, 49, 50, 51, 52, 53, 54, 55^

### Light-filtering properties of the non-cataractous crystalline lens

The crystalline lens blocks UV radiation between 300 and 400 nm, up to 390 nm in a young eye and up to 400 nm in a 63-year-old lens.^9, 56^ The human lens grows throughout an individual's lifespan by a process of epithelial cell division, yet none of its cells are cast off. Such terminal differentiation within a closed avascular system necessitates continuous remodelling to achieve such growth and maintain light transmission.^11^ However, the cumulative insults of radiation, oxidation, and post-translational modification result in an age-related increase in light scatter, fluorescence, and spectral absorption, especially at the short-wavelength end of the visible spectrum. The greatest increase in absorption is for wavelengths ~460–470 nm (blue light), and this is largely attributable to post-natal accumulation of chromophores.^57^

### Light-filtering properties of the cataractous crystalline lens

The aging, yellowing, and opacified crystalline lens is only one-third as translucent to visible short-wavelength light when compared with the youthful lens. For example, a 53-year-old and a 75-year-old crystalline lens transmit 70 and 25% of incident visible blue light, respectively.^58^

### Light-filtering properties of UV-only filtering IOLs

Initially, IOLs were manufactured from polymethylmethacrylate and did not include UVR-blocking chromophores.^59^ Retinophototoxicity attributable to UVR transmission was recognized in 1978,^59, 60^ such that most IOLs contained UVR-absorbing chromophores by 1980.^61^

### Visible light-filtering IOLs

**Figure 3:**
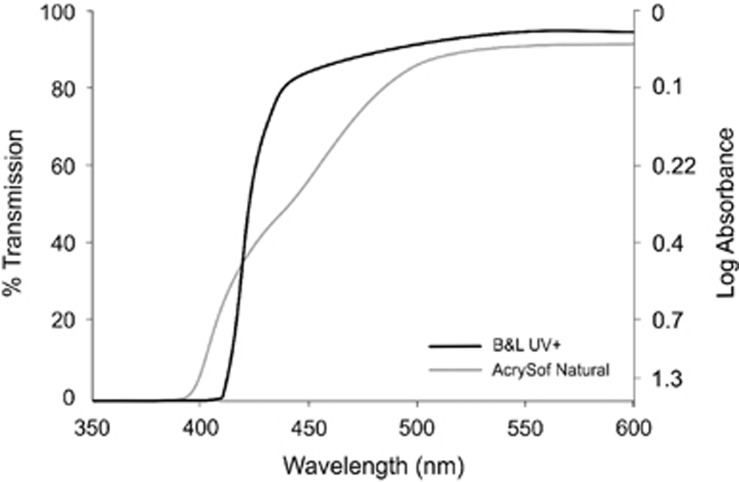
Comparison of the different transmission properties of Alcon AcyrSof Natural (blue light-filtering IOLs) and Bausch + Lomb SofPort AO with Violet Shield Technology (UV-only filtering IOLs).^7^

A standard UV-only filtering IOL typically absorbs wavelengths up to 420 nm. In contrast, blue light-filtering IOLs contain chromophores that block wavelengths between 400 and 500 nm, and are typically classed as blue-blockers (absorbing visible light in the blue part of the spectrum, circa 450–500 nm) and violet-blockers (absorbing only the violet part of the spectrum, circa 410–440 nm, but transmitting blue light). For example, the Alcon AcrySof Natural IOL reduces transmittance of short-wavelength light by 94% (1.22 log units of absorbance) at 400 nm, 53% (0.33 log units of absorbance) at 450 nm, and 31% (0.16 log units of absorbance) at 475 nm, and is therefore considered a blue light-filtering IOL.^62^ The Bausch + Lomb SofPort AO with Violet Shield Technology reduces short-wavelength light transmission by 99.5% (2.3 log units of absorbance) at 400 nm, 36.8% (0.2 log units of absorbance) at 425 nm, 11.2% (0.05 log units of absorbance) at 450 nm, and 9.2% (0.04 log units of absorbance) at 475 nm, and is therefore considered a violet blocker^7^ (Figure 3).

## **The rationale and evidence base upon which implantation of blue-light-filtering iols is premised**

### Blue-light-filtering IOLs and central vision

A patient-masked, randomized, crossover study (evidence level 2b, Table 1), involving 154 bilaterally pseudophakic patients (having been implanted with UV-only filtering IOLs) were recruited to investigate whether filtration of visible short-wavelength blue light impacted on visual performance and experience under conditions of intense light (0.1 candela/m^2^).^63^ Outcome measures included photostress recovery time and glare disability thresholds, under conditions of wearing clip-on blue light-filtering spectacles *vs* wearing clip-on UV-only filtering spectacles. In brief, the use of a clip-on blue-light-filtering lens on pseudophakic patients increased their ability to tolerate glare and enhanced their recovery following photostress. However, it should be noted that these results can only give an indication as to whether there is a difference between the use of blue light-filtering clip-on lenses *vs* UV-only filtering clip-on lenses on already pseudophakic eyes and, even then, only under conditions of intense light.

A non-randomized, controlled trial of Alcon AcrySof Natural IOLs (blue light-filtering IOLs) *vs* Alcon AcrySof single piece IOLs (UV-only filtering IOLs), representing level 3b evidence and including one eye (the study eye) of 93 patients in whom the fellow eye was already pseudophakic with a UV-only filtering IOL, revealed no difference between the two IOL types in terms of visual acuity (VA), CS or color perception.^64^

In a comparative, single center, non-randomized study (evidence level 3b, Table 1), 60 eyes of 30 patients were implanted with either a UV-only filtering IOL (AcrySof SA60AT) in one eye or a blue light-filtering IOL (AcrySof SN60WF) in the fellow eye.^10^ Two years following surgery, VA, color vision, CS, macular thickness, and macular volume were compared, and there were no significant differences between fellow eyes in terms of any of these outcome measures.^10^

A retrospective study (evidence level 3b, Table 1) designed to compare the prevalence of cyanopsia (seeing everything tinged with blue) in eyes implanted with either a UV-only filtering IOL or a blue light-filtering IOL,^65^ where cyanopsia was graded on the basis of white gradation cards (in a system known as the neutralization method), has been reported. The study comprised one eye of patients implanted with a UV-only filtering IOL (Acrysof SA60AT; *n*=57), known as Group 1, and one eye (of different patients) implanted with the blue light-filtering IOL (Hoya AF-1 YA60BB; *n*=96), known as Group 2, and a further reference group (*n*=41) comprising healthy patients without either cataract or prior cataract surgery.^65^ Although cyanopsia did occur more frequently in Group 1 than in Group 2 one month postoperatively (14.5 *vs* 4.9% *P*=0.049), this difference was no longer significant 3 months following surgery (9.1 *vs* 5.2% *P*>0.05).

One small randomized study (evidence level 2b, Table 1) demonstrated superior CS postoperatively in association with use of blue light-filtering IOLs *vs* UV-only filtering IOLs.^66^ Sixty patients scheduled for cataract surgery were randomly assigned to receive either a blue light-filtering IOL (model not specified; *n*=30) or a UV-only filtering IOL (model not specified; *n*=30) in one eye, where the fellow eye was cataractous. VA, CS, and color vision were examined up to 6 months postoperatively, when no significant difference in VA or color vision between eyes implanted with a UV-only filtering IOL *vs* a blue light-filtering IOL was observed, although superior CS at select spatial frequencies was noted among eyes implanted with a blue light-filtering IOL one week postoperatively (1.5 c/d, *P*=0.0076; 3 c/d, *P*=0.0142; and 6 c/d, *P*=0.0269), 1 month postoperatively (1.5 c/d, *P*=0.0067; 3 c/d, *P*=0.0088; and 6 c/d, *P*=0.0098), 3 months postoperatively (1.5 c/d, *P*=0.0047; 3 c/d, *P*=0.0051; and 6 c/d, *P*=0.0033), and 6 months postoperativerly (1.5 c/d, *P*=0.0058; 3 c/d, *P*=0.0046; and 6 c/d, *P*=0.0026).^66^ However, this study is fundamentally flawed as the authors did not measure or report preoperative measures of CS in either group, and post-operative disparity between the two IOL groups may simply reflect preoperatively disparity between the two groups in this respect. Other obvious shortcomings in this study include: cataract operations were performed by extracapsular cataract surgery; the failure to specify the models of IOLs implanted; the failure to measure and control for MP (the augmentation of which is now known to enhance CS in diseased^42, 43, 44, 45^ and non-diseased eyes^34, 46, 47, 48, 49, 51, 52, 53^). Accordingly, it is doubtful whether this study should, in fact, be classed as level 2b.

### Blue-light-filtering IOLs and photopic vision

In one study (evidence level 2b, Table 1), one eye of 98 patients was randomly implanted with either a UV-only filtering IOL (AcrySof SA60AT) or a blue light-filtering IOL (AcrySof SN60AT with IMPRUV filter).^67^ One month following surgery, CS was measured under photopic and mesopic conditions in the study eye, and it was observed that eyes implanted with blue light-filtering IOLs were comparable to those implanted with UV-only filtering IOLs in terms of the outcome measures (VA, photopic CS and mesopic CS). An obvious shortcoming of this study rests on the minuscule duration of follow-up for the intended purpose.

Importantly, it should be reiterated that none of the studies comparing central cone-mediated, visual function following implantation of blue light-filtering IOLs *vs* UV-only filtering IOLs have measured (or corrected for) MP, the inter-individual variability of which is substantial,^54, 68^ and are therefore only in a position to comment on the impact of a blue-light filter (the blue light-filtering IOL) superimposed on another (but unmeasured) blue-light filter (ie, MP).^58, 64, 66^

### Blue-light-filtering IOLs and mesopic/scotopic central and peripheral vision

**Figure 4:**
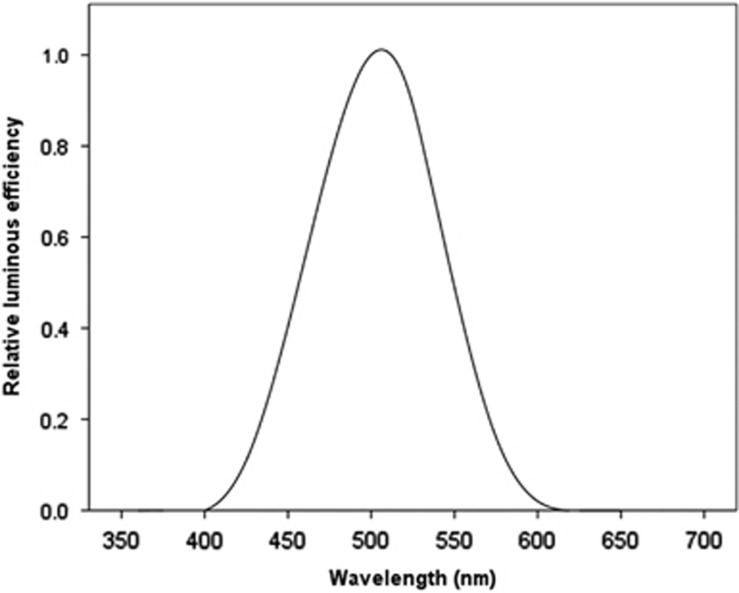
The relative spectral luminous scotopic efficiency of the CIE standard photometric observer (V′*_λ_*) depends on short-wavelength visible (blue) light and peaks at 507 nm.^69, 70^

For an eye adapted to dim illumination, scotopic sensitivity peaks at 505 nm in the blue-green part of the spectrum, and cannot detect wavelengths greater than about 640 nm.^12, 40^ The Commission Internationale de l'Eclairage (CIE) standard spectral luminance efficiency function for scotopic vision, known as V'_λ_, and illustrated in Figure 4, is dependent on short-wavelength visible (blue) light and is similar to the absorption spectrum for rhodopsin (which peaks at 500 nm).^69, 70^ Indeed, that observation represented the key reason for concluding that rhodopsin, which is only contained in rods, mediates scotopic vision.^71, 72^

**Figure 5:**
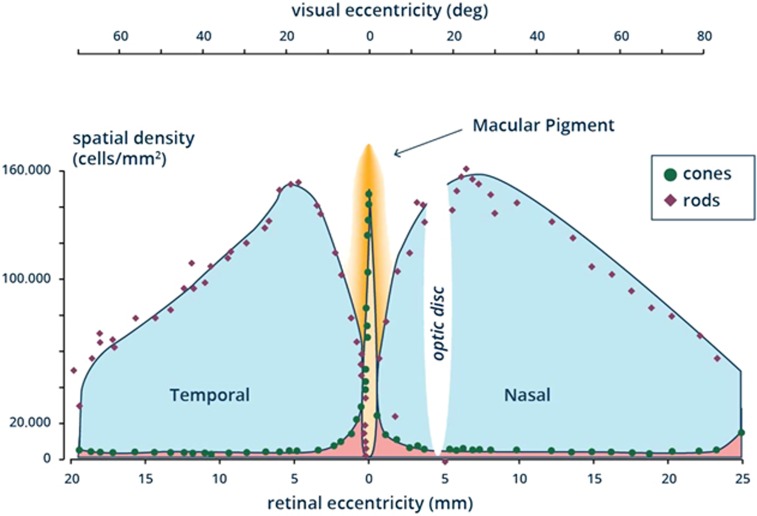
Illustration of distribution of the photoreceptors (cones and rods) across the retina.^73, 75^

There are ~91 million rod photoreceptors and 4.5 million cone photoreceptors in the human retina.^73^ There are no rods contained in the central 1.25° of the fovea,^74^ whereas cone density peaks at this location (100 000–324 000 cones/mm^2^, but subject to considerable inter-individual variability) and declines to 5000 cones/mm^2^ or less in peripheral retina^39, 75^ (Figure 5).

Photopic and scotopic sensitivity decline with increasing age. However, the rate of age-related decline in scotopic vision is twice as fast as that of photopic vision, and results in the observed difficulties that older adults experience in dim environments.^76^ The reason for the faster age-related decline in scotopic vision is probably related to the observation that aging has little effect on the number of human foveal cone photoreceptors, whereas parafoveal rod photoreceptors decline in number by 30% with increasing age.^77, 78^

Specifically, scotopic CS declines at low and high spatial frequencies,^79^ and rod-mediated dark adaptation slows progressively with increasing age.^80^ Further, the age-related loss of scotopic sensitivity is most severe for visible short-wavelength (blue) light.^81^ These age-related changes in scotopic visual function contribute to an increased risk of falling,^82^ and the need to be closer to road signs to read them effectively at night,^83^ amongst older adults.

The Purkinje shift, characterized by the differing peaks of spectral sensitivity for scotopic (507 nm) and photopic (555 nm) vision, results in blue light being responsible for 7% of photopic vision but for 35% of aphakic scotopic vision.^13, 14^ As blue light-filtering IOLs filter out 27–40% of blue light between 440 and 500 nm, compared with only 6% of such wavelengths being filtered out by standard UV-only filtering IOLs, there is a real concern that IOLs that filter short-wavelength visible light selectively and adversely impact upon the ability to see in the dark.^84^ Theoretical calculations estimate that blue light-filtering IOLs reduce scotopic sensitivity by 14–21%, depending on dioptric power.^84, 85^

Commentators who have argued that the observed decrease in scotopic retinal sensitivity attributable to blue light-filtering IOLs is not clinically meaningful have premised their views on the fact that blue visible light represents only a small portion of the entire visible spectrum,^86^ and that such blue light-filtering IOLs show similar transmittance to a 75-year-old lens.^58^ However, and given that the primary objective of cataract surgery is to restore pre-cataract vision and to prevent further age-related decline in vision, it is difficult to argue in favour of replacing an aging, yellowing and cataractous lens with a manufactured IOL with similar absorbance characteristics, which cannot in any way alleviate the age-related decline in scotopic vision that older adults find so disturbing. Indeed, it is reasonable to hypothesise that the ability to accumulate the macular carotenoids only in the central retina has evolved to optimize the quality of central vision by attenuating the adverse effects of (blue) light scatter and CA, whilst ensuring that the non-central retina is not deprived of visible short-wavelength light that is so important for vision under dim light.^87^

In other words, blue light-filtering IOLs necessarily filter blue light, which is responsible for 35% of aphakic scotopic vision, and such IOLs may necessarily and adversely impact on vision under dim light conditions, but are unlikely to adversely impact on vision mediated (even in part) by cones (ie, photopic and mesopic vision), because the filtered visible wavelengths are responsible for only 7% of photopic vision. To test the hypothesis that vision mediated solely or partly by rods (ie, scotopic and mesopic vision, respectively) is adversely affected by implantation of blue light-filtering IOLs, vision needs to be tested in subjects with such IOLs *vs* subjects with implanted standard UV-only filtering IOLs, but in a way that takes full account of the impact of another prereceptoral blue light filter (ie, MP) and to test mesopic and scotopic retinal sensitivity where there is no MP (ie, beyond 7° eccentricity). Unfortunately, however, there is not a single study attempting to investigate the impact of blue light-filtering IOLs on central vision where MP was measured, and only a solitary study has measured scotopic retinal sensitivity in retina devoid of MP, where it was confirmed that such IOLs did indeed adversely impact on rod-mediated retinal sensitivity.^88^

### Studies investigating mesopic/scotopic vision following implantation of blue-light-filtering IOLs

The vision in darkness that is mediated solely by rods is known as scotopic vision. In one paper (evidence level 3b, Table 1), 22 patients with bilateral pseudophakia and early AMD were recruited to investigate whether a blue light-filtering IOL would affect vision-dependent tasks under scotopic conditions.^89^ These vision-dependent tasks, which included tests of eye-hand coordination and of mobility, were performed with and without blue-light-filtering spectacles (worn in randomized order), and challenges included a mobility obstacle course, manipulation of cylindrical blocks and a psychophysical dark-adapted full-field flash test. Further, a navy *vs* blue sock color-sorting task was used to evaluate photopic color discrimination. Although performance of these tasks under scotopic conditions did not differ when patients attempted to conduct them with or without blue-light-filtering spectacles, failure to discriminate between navy *vs* blue sock in the color-sorting task was significantly higher amongst those wearing blue-light-filtering spectacles (*P*<0.001).

Greenstein *et al*^88^ reported another level 3b study designed to investigate possible adverse effects of a blue-light-filtering IOL on scotopic sensitivity and hue discrimination under scotopic conditions. Nine patients with a blue light-filtering IOL (AcrySof SN60AT) in one eye and a UV-only filtering IOL (AcrySof SA60AT) in the fellow eye, as well as another nine young phakic patients (tested under conditions with a yellow-tinted clip-on lens and without such a lens) were recruited for the purposes of this study. Hue discrimination and dark-adapted thresholds to 440, 500, and 650 nm light were measured, and results suggested that, in the 9 operated patients, there were no significant differences in hue discrimination or dark-adapted sensitivity between fellow eyes.^88^ However, with the clip-on lens, mean sensitivities to the 440, 500, and 650 nm stimuli were significantly decreased by 2.7–2.8 dB, 0.7–1.0 dB, and 0–1.2 dB, respectively.^88^

Another level 3b study designed to compare measures of photopic and scotopic CS in eyes with an AcrySof SN60AT Natural IOL (blue-light-filtering) and eyes with a conventional AcrySof SA60AT IOL (UV-only filtering) was reported, where the right eye of 38 patients was implanted with the former and right eye of 38 age-matched controls with the latter.^90^ In brief, no statistically significant differences were observed between the two IOL types in terms of CS, scotopic CS or in terms of blue-green color vision.

In the study (level 2b) reported by Kara-Junior,^91^ where one eye of 60 subjects was implanted with a UV-only filtering IOL and the fellow eye implanted with a blue-light-filtering IOL, there was no difference between the two IOL types in terms of CS or color vision; further, a detailed examination, which included measurement of central retinal thickness by optical coherence tomography and a judgement on whether clinically significant macular change were apparent on slit-lamp biomicroscopy, was performed by a retinal specialist 5 years after surgery, and there was no difference between the two IOL types in this respect.

There is a weakness inherent in all studies that have attempted to investigate mesopic visual function following implantation of blue light-filtering IOLs, namely that all have utilized measures that are a function of only central vision, where MP is also acting as a blue light filter (but MP was not measured or corrected for in any of these studies). Further, of the four studies reporting on ‘scotopic' vision in eyes following implantation with blue-light-filtering IOLs,^88, 90, 91, 92^ two used luminance values >1 cd/m^2^,^91, 92^ thereby, in fact, reporting on mesopic vision that is mediated, at least in part, by cones, and therefore less likely to be adversely influenced by the transmittance properties of such blue-blocking IOLs.

### Blue-light-filtering IOLs and non-vision-forming perception

In higher animals, photoreception can be classed as vision-forming and non-vision-forming, and in mammals the former is mediated by rods and cones, whereas the latter is mediated by the intrinsically photosensitive retinal ganglion cells (ipRGCs), which contain melanopsin.^93^ Indeed, the detection of irradiation by melanopsin stimulates photoentrainment of circadian rhythm via the retinohypothalamic tract, in a way that can convey non-vision-forming perception in the absence of functioning rods and cones (although the output of ipRGCs is normally regulated by input from these photoreceptors).^94, 95^ The maintenance of photoentrainment of circadian rhythm in persons with no conscious perception of light is attributable to ipRGCs.^96^

It has been demonstrated that exposure to blue light can suppress melatonin secretion, thereby linking photoreception with sleep regulation.^97, 98^ Indeed, Herljevic *et al*^99^ have shown significantly reduced melatonin suppression in elderly subjects following exposure to short-wavelength visible light (456 nm) compared with young subjects, and attributed their findings to the transmittance properties of cataracts. Melanopsin (a photosensitive pigment contained within ipRGCs) is maximally stimulated by blue light at ~480 nm, with significant absorption down to 420 nm,^100, 101^ prompting concern that implantation of blue-light-filtering IOLs could contribute to sleep disorders.^101^ Some commentators have estimated that blue-light-filtering IOLs are associated with 27–38% less melatonin suppression than standard UV-only filtering IOLs, although comparisons with a cataractous lens have not been made.^14^ Given that, in older individuals, the effective retinal light exposure is circa one-tenth that of younger individuals,^102^ and since cataract surgery can therefore be expected to greatly increase melatonin suppression, it is unlikely that implantation of a blue light-filtering IOL to replace a yellow cataract would have a disproportionate impact on circadian cycles. Indeed, this view is borne out by studies demonstrating improved sleep patterns following cataract surgery, in a way that is comparable for patients in whom blue-light-filtering IOLs were implanted *vs* those in whom standard UV-only IOLs were implanted.^103, 104^

Ayaki *et al*^105^ reported a study (71 patients; mean (SD) age, 74.1 (±8.8)) on the subject of sleep pattern following cataract surgery using UV-only filtering IOLs (evidence level 3b, Table 1), and concluded that implantation of an UV-only filtering IOL has potential for improving sleep quality and gait speed, in addition to restoring vision and vision-related quality of life. Indeed, improved sleep patterns have consistently been reported following cataract surgery where UV-only filtering IOLs were implanted at the time of procedure.^106, 107, 108^

In one cohort study (evidence level 4, Table 1), 206 patients undergoing cataract surgery with implantation of a UV-only filtering IOL (Acrysof SA60AT) or a blue light-filtering IOL (Acrysof SN60WF) were recruited to make comparisons in terms of post-operative quality of life and sleep patterns.^109^ The outcome measures were evaluated using the National Eye Institute Visual Function Questionnaire (VFQ-25) and Pittsburgh Sleep Quality Index (PSQI) before surgery, and again 2 months and 7 months post-operatively. In brief, there were significant improvements in the sub-scale scores for sleep latency (*P*<0.001 at 7 months, unpaired *t* test) and sleep disturbance (*P*<0.05 and *P*<0.01 at 2 and 7 months, respectively) in patients implanted with a UV-only filtering IOL, but no such improvements were noted for eyes implanted with the blue-light-filtering IOL.^109^

A single center, double-masked, block-randomized clinical trial (evidence level 2b) involving one eye (the eye with lowest visual acuity) of 76 patients was reported to investigate the effect of cataract surgery on circadian photoentrainment, and to see if there was any difference between blue-light-filtering IOLs and UV-only filtering IOLs in this respect.^110^ The primary outcome measure was activation of intrinsic photosensitive ganglion cells (using post-illumination pupil response to blue light from 10 to 30 s after light exposure), which was measured 2 days and 3 weeks following the procedure. Secondary outcomes included circadian rhythm and measures of 24-h salivary melatonin, where sleep quality was determined by actigraphy and the Pittsburgh Sleep Quality Index. In brief, the authors observed no difference between patients who received blue light-filtering IOLs *vs* the UV-only filtering IOLs, but the authors conceded that the study was not designed to address the possible impact of cataract surgery on circadian photoentrainment or sleep in the longer term or following surgery in the second (fellow) eye.^110^

In one non-randomized study (evidence level 4, Table 1), 961 patients were implanted with either a UV-only filtering IOL (*n*=498) or a blue light-filtering IOL (*n*=463) at the time of cataract surgery.^104^ PSQI scores were recorded 1, 6, and 12 months following the surgery, and, in brief, overall sleep quality and sleep latency improved following the procedure, irrespective of the absorbance properties of the implanted IOLs (and there was no observed difference between the two IOLs).^104^

In another study (evidence level 2b, Table 1), 80 patients were randomised to receive either a UV-only filtering IOL (AMO Tecnis ZCB00) or a blue light-filtering IOL (Acrysof SN60WF) in at least one of two eyes.^111^ Outcome measures included reaction time (response to sensory stimulus) and the Epworth Sleepiness Score (ESS) questionnaire. It was found that cataract surgery (particularly first-eye surgery) had a beneficial effect in terms of cognition and daytime alertness, regardless of the absorbance properties of the implanted IOL (and there was no observed difference between the two types of IOL).^111^

A large-scale study was designed to investigate changes in blood pressure and sleep duration following cataract surgery with IOL implantation (various models, some blue light-filtering (*n*=1059) and some not (*n*=308)), and to investigate how different types of IOL influence the degree of observed effects (evidence level 3b).^112^ Information relating to sleep duration (6 h or less, between 6.5 and 8 h, or 8.5 h or more) were collected from 1367 patients (who were scheduled to and subsequently underwent cataract surgery in one eye) before surgery, 1 week after surgery and 1 month after surgery, and it was reported that sleep duration was improved following cataract surgery, irrespective of the types of IOL implanted at the time of the procedure.^112^

### Blue-light-filtering IOLs and phototoxicity

Oxidative stress refers to tissue damage by unstable molecules, known as reactive oxygen intermediates (ROIs), and these compounds include free radicals, hydrogen peroxide, and singlet oxygen.^113^ The retina is especially susceptible to oxidative stress as it has the highest oxygen metabolism in the mammalian world, and because it is exposed to irradiation (ie, visible light), which is known to increase production of ROIs. Of the visible spectrum, high-energy short-wavelength visible light is the most injurious in terms of ROI production.^113^ Further, the retina is particularly vulnerable to damage by ROIs because of the high concentration of poly-unsaturated fatty acids (PUFAs) in the outer segment membranes of the photoreceptors, as PUFAs contain readily accessible electrons in their double bonds. As a consequence, it has been hypothesized that oxidative stress and cumulative lifetime exposure to visible light are important factors in the pathogenesis of AMD, and this hypothesis is consistent with the proven benefits of antioxidant supplements in terms of retardation of disease progression.^114, 115^

Valid studies designed to investigate the relative importance of retinal exposure to short-wavelength visible (blue) light following cataract surgery are unlikely to yield definitive results, as it would be impossible to control for the cumulative exposure to such visible wavelengths before surgery, which would be dependent on a plethora of variables including the age of the patient at the time of surgery, the duration and extent of the yellowing of the lens opacity, MPOD, amongst other factors. In other words, the hypothesized photoprotective benefits of implanting blue-light-filtering IOLs at the time of cataract surgery is unlikely to be either proven or refuted, and the surgeon must therefore elect to make a decision based on a rationale rather than on an evidence base. Of note, implantation of UV-only filtering IOLs at the time of cataract surgery in 1167 of 4577 AREDS participants with intermediate age-related macular degeneration (AMD), in the context of 6 monthly retinal reviews and follow-up of at least 5 years, was not associated with progression to advanced AMD.^116^

A 2-year prospective observational study (evidence level 4, Table 1) was reported by Nagai *et al*^117^, and was designed to evaluate changes in fundus autofluorescence in the 2 years following implantation of blue-light-filtering IOLs (YA-60BBR, Hoya Corp.) *vs* UV-only filtering IOLs (VA-60BBR, Hoya Corp.) at the time of cataract surgery. Abnormal fundus autofluorescence has been recognized as predictive of development of geographic atrophy and neovascular AMD.^118, 119^ The study consisted of 52 eyes of 52 patients where a blue-light-filtering IOL was implanted and 79 eyes of 79 patients where a UV-only filtering IOL was implanted, and outcomes included changes in fundus autofluorescence and evidence of AMD development and, in particular, geographic atrophy. In this study, increased fundus autofluorescence was seen in the UV-only filtering IOL group only, suggesting that the blue-light-filtering IOL might protect against AMD. The limitations of this study include its small number of subjects and its design. A comparative randomized study with more patients and a longer period of follow-up is required to investigate whether or not blue light-filtering IOLs are protective against AMD development. Another criticism of this paper rests on the fact that it is very likely that the measures of autofluorescence are influenced by the absorbance properties of the preoperative cataract and of the implanted IOL^120^ and, accordingly, the severity and grade of the preoperative lens opacity and the impact of the IOL's absorbance properties on measurements should be factored into analysis.

In another study, representing evidence level 3b (Table 1), the impact of the filtration properties of the implanted IOL on progression of geographic atrophy was assessed, where 66 eyes of 40 patients afflicted with AMD were implanted with either a blue light-filtering IOL (*n*=27; AcrySof SN60WF) or a UV-only filtering IOL (*n*=39; various models), and followed for a period of 1 year following surgery.^121^ The primary outcome measure was progression of geographic atrophy, and the data suggested a protective effect in association with implantation of blue-light-filtering IOLs.^121^ However, it should be noted that there are many variables affecting rates of progression of atrophic AMD, including age and genetic background, and these were not factored into the analysis;^122, 123, 124^ further, it is very likely that measures of autofluorescence are profoundly influenced by the nature and density of the cataract before surgery (not reported in this study) and by the absorbance properties of the IOL, thus confounding pre and post-operative measures of autofluorescence and negating any meaningful discussion.

## **A** comment on violet-blocking iols

The term ‘violet-blocking' IOL has been coined to describe IOLs that block wavelengths between 400 and 440 nm but do not block wavelengths >440 nm,^14^ and such IOLs have been introduced in an attempt to limit the disadvantages inherent in depriving the non-central retina of wavelengths between 440 and 500 nm. However, and to our knowledge, there have been no studies comparing violet-blocking IOLs with blue-blocking IOLs in terms of vision-forming or non-vision-forming outcomes, or in terms of photoprotection.

## **Conclusion**

In terms of photoprotection, there is no level 2b (or higher) evidence in support of using blue light-filtering IOLs *vs* UV-only filtering IOLs at the time of cataract surgery. In general, the quality of evidence informing the surgeon's selection of IOLs on the basis of light transmittance properties is deficient.

## Method of literature search

References for this review were identified through a retrospective literature search of the electronic PubMed database (2000–2016), and included where appropriate. The following key words and combination of these words were used in compiling the search: blue blocker, blue light-filtering, lens, AcrySof Natural, SN60AT and YA60BB. We included all studies involving patients (both male and female, above 16 years of age) undergoing cataract surgery and implantation of blue light-filtering IOLs.

## Figures and Tables

**Table 1:** Evidence levels

*Principal author*	*Level of evidence*	*Subjects*	*Study eyes*	*Study design*	*Main outcome measures*	*Financial support*	*Advocates*	*Comment*
*Vision as the primary outcome measure*								
Landers *et al*^64^	3b	93	93	C	VA and CS	No	No	
Yuan *et al*^66^	2b	60	60	C	CS and color vision	Not declared	Yes	Small size RCT
Greenstein *et al*^88^	3b	18	36	A, D	Hue discrimination	Alcon lab.	Yes	
Muftuoglu *et al*^90^	3b	76	76	C	CS and blue-green discrimination	No	No	
Schmack *et al*^91^	2b	22	44	A	VA, color discrimination, and CS	No	No	Small size RCT
Kara-Junior *et al*^92^	2b	30	60	A	CS, color version, and macular morphology	No	No	Small size RCT
Lavric *et al*^10^	3b	30	60	A	VA, CS, color vision, and macular test	No	No	
Bandyopadhyay *et al*^67^	2b	98	98	C	VA, photopic CS, and mesopic CS	No	No	Short term RCT
Miyata^65^	3b	194	194	C	Cyanopsia	No	Yes	
								
*Vision as the primary outcome measure (but cataract surgery was not performed for the purpose of the study)*								
Hammond^63^	2b	154	154	D	Photostres recovery time	Alcon res.	Yes	Short term RCT
Kiser *et al*^89^	3b	22	22	D	VA and color discrimiation	Not declared	No	
*Principal author*	*Level of evidence*	*Subjects*	*Study eyes*	*Surgery type*	*Main outcome measures*	*Financial support*	*Advocates*	*Comment*
*Sleep pattern as the primary outcome measure*								
Landers *et al*^103^	4	49	98	B	PSQI	No	Yes	
Alexander *et al*^104^	4	961	961	C	PSQI	No	No	
Asplund *et al*^106^	4	328	328	C	Questionnaire	Not declared	No	
Asplund *et al*^107^	4	407	407	C	Questionnaire	Not declared	No	
Brondsted *et al*^110^	2b	76	76	C	PSQI	No	No	Short term RCT
Ayaki *et al*^105^	3b	71	142	B	VFQ-25, PSQI, and gait speed	No	No	
Ayaki *et al*^109^	4	206	412	B	VFQ-25 and PSQI	No	No	
Ichikawa *et al*^112^	3b	1367	1367	C	Blood pressure and sleep duration	No	Yes	
Schmoll *et al*^111^	2b	80	160	B	Reaction time and ESS	No	No	Small size RCT
								
*Progression of AMD and/or macular morphology*								
Chew *et al*^116^	2b	4577	8050	B	Geographic atrophy and AMD	No	No	
Nagai *et al*^117^	4	131	131	C	Fundus autofluorescence	No	Yes	
Pipis *et al*^121^	3b	40	66	A, C	Geographic atrophy	No	Yes	
Kara-Junior *et al*^92^	2b	30	60	A	CS, color vision, and macular morphology	No	No	Small size RCT

Abbreviations: advocates, advocates for use of blue light-filtering IOLs; ESS, Epworth Sleepiness Score; financial support, financial support received from manufacturers/distributors of blue light-filtering IOLs; PSQI, Pittsburgh Sleep Quality Index; study design, surgery type A: both eyes operated on for the purpose of the study, where one eye was implanted with a UV-only filtering IOL and the fellow eye implanted with a blue-light-filtering IOL; B: both eyes operated on for the purpose of the study, where at least one eye was implanted with either a blue light-filtering IOL or a UV-only filtering IOL; C: only one of two eyes operated upon for the purpose of the study, where the fellow eye may has been either pseudophakic or cataractous; D: no surgery performed for the purpose of the study; VFQ-25, Visual Function Questionnaire.
